# Development of p-Tau Differentiated Cell Model of Alzheimer’s Disease to Screen Novel Acetylcholinesterase Inhibitors

**DOI:** 10.3390/ijms232314794

**Published:** 2022-11-26

**Authors:** Giuseppe Uras, Xinuo Li, Alessia Manca, Antonella Pantaleo, Marco Bo, Jinyi Xu, Stephanie Allen, Zheying Zhu

**Affiliations:** 1 School of Pharmacy, The University of Nottingham, University Park, Nottingham NG7 2RD, UK; 2 Department of Clinical and Movement Neurosciences, Institute of Neurology, University College London, London NW3 2PF, UK; 3 School of Pharmacy, China Pharmaceutical University, Nanjing 210009, China; 4 Department of Biomedical Sciences, University of Sassari, Viale San Pietro 43b, 07100 Sassari, Italy

**Keywords:** Alzheimer, AChE, GSK3-β, inhibitors, Tau, hyperphosphorylation

## Abstract

Alzheimer’s disease (AD) is characterized by an initial accumulation of amyloid plaques and neurofibrillary tangles, along with the depletion of cholinergic markers. The currently available therapies for AD do not present any disease-modifying effects, with the available in vitro platforms to study either AD drug candidates or basic biology not fully recapitulating the main features of the disease or being extremely costly, such as iPSC-derived neurons. In the present work, we developed and validated a novel cell-based AD model featuring Tau hyperphosphorylation and degenerative neuronal morphology. Using the model, we evaluated the efficacy of three different groups of newly synthesized acetylcholinesterase (AChE) inhibitors, along with a new dual acetylcholinesterase/glycogen synthase kinase 3 inhibitor, as potential AD treatment on differentiated SH-SY5Y cells treated with glyceraldehyde to induce Tau hyperphosphorylation, and subsequently neurite degeneration and cell death. Testing of such compounds on the newly developed model revealed an overall improvement of the induced defects by inhibition of AChE alone, showing a reduction of S396 aberrant phosphorylation along with a moderate amelioration of the neuron-like morphology. Finally, simultaneous AChE/GSK3 inhibition further enhanced the limited effects observed by AChE inhibition alone, resulting in an improvement of all the key parameters, such as cell viability, morphology, and Tau abnormal phosphorylation.

## 1. Introduction

Alzheimer’s disease (AD) is a neurodegenerative disease, currently recognized as the worldwide leading cause of dementia, reportedly affecting more than 50 million patients in the world, with this figure expected to be at least doubled by 2050 [1]. The disease is characterized by an array of cognitive symptoms, such as memory impairment, learning disabilities, and behavioral changes, which lead to a progressive loss of independency in routine tasks by the affected patients [2]. The typical pathology is presented by a severe progressive neurodegeneration, accumulation of amyloid plaques, and presence of neurofibrillary tangles (NFTs) [3].

NFTs are found in the neuronal cytoplasm of AD patients and are composed of abnormally phosphorylated Tau protein. Tau hyperphosphorylation is the result of the aberrant activation of either kinases or phosphatases. One of the most studied kinases, responsible for the phosphorylation on Tau protein at multiple residues, is GSK3-β. The latter is ubiquitously expressed in the central nervous system (CNS), and is activated by a number of different pathways, which are often linked to AD [4,5,6,7,8]. Moreover, phosphorylation performed by GSK-3-β on Tau results in a loss of affinity to microtubules and promotion of Tau self-aggregation [9,10]. In AD, as well as in other tauopathies, the aberrant phosphorylation process that occurs on Tau triggers its assembly in paired helical filaments (PHF) and straight filaments (SF), which leads to the development of NFTs [11].

It has been also shown that hyperphosphorylated Tau is required to trigger amyloid toxicity in vivo [12,13].

Multiple factors are involved in the triggering of Tau hyperphosphorylation, such as amyloid plaques, glucose metabolism impairment, and RAGE-pathway activation [14,15,16]. Despite being through different pathways, all factors involved in altering Tau phosphorylation levels lead to the activation of specific kinases enzymes or the inhibition of phosphatases enzymes [17].

In the present study, we have focused on GSK3-β as a potential target for AD therapy, which is responsible for phosphorylating Tau on residue S396, in which abnormal phosphorylation is considered to be an early marker of subsequent hyperphosphorylation [18]. GSK3-β is the downstream kinase activated by multiple pathways linked to Tau hyperphosphorylation [5,18,19,20,21]. Thus, targeting GSK3-β could result in the inhibition of Tau phosphorylation induced by multiple pathways in AD, and, therefore, block the disease progression.

The complexity of AD has frequently limited the realism of experimental models, which often results in contradictive results. Cell line SH-SY5Y has often been used as a cell culture model of AD, in particular for evaluating AChE inhibitors due to its expression of AChE enzyme in cells [22,23,24,25], despite bearing the crucial limitation of being constantly dividing. However, several approaches have been proposed to further improve the SH-SY5Y cell model by using retinoic acid (RA) as a differentiation factor, and foetal bovine serum (FBS) deprivation to stop the constant division [26,27,28,29,30]. Moreover, a number of methods exploiting undifferentiated SH-SY5Y modelled Tau hyperphosphorylation through different mechanisms, such as hypothermia, okadaic acid administration, and glyceraldehyde (GA) exposure [31,32,33]. The usage of GA to induce Tau hyperphosphorylation is of peculiar interest, since GA treatment leads to increased levels of advanced glycated end products (AGEs), and the receptor, RAGE, activates an important and well-studied pathway in GSK3-β activation [20,21,33].

To study hyperphosphorylation mechanisms of Tau we have developed and validated a new cell-based AD model by differentiating SH-SY5Y into mature neurons featuring morphological and p-Tau characteristics with administration of GA to trigger the Tau aberrant phosphorylation [26,33]. Finally, we exploited such a model to assess the efficacy of novel single- or dual-targeting acetylcholinesterase (AChE) inhibitors; the core structure of these new drug candidates is derived from the (±)-7,8-Dihydroxy-3-methylisochroman-4-one [(±)-XJP] compound, which was initially isolated from the *Musa sapientum* L., with a 4-F as R group and named XJP-1 [34,35,36]. In addition to this, given the relevance and central role of the GSK3-β enzyme in multiple pathways during AD pathogenesis, a novel dual AChE/GSK3-β inhibitor named **27g** has been tested (Table 1) [37].

## 2. Results

### 2.1. GA Treatment Induces Abnormal Tau Phosphorylation and Axon Degeneration in SH-SY5Y-Differentiated Neurons

**Figure 1:**
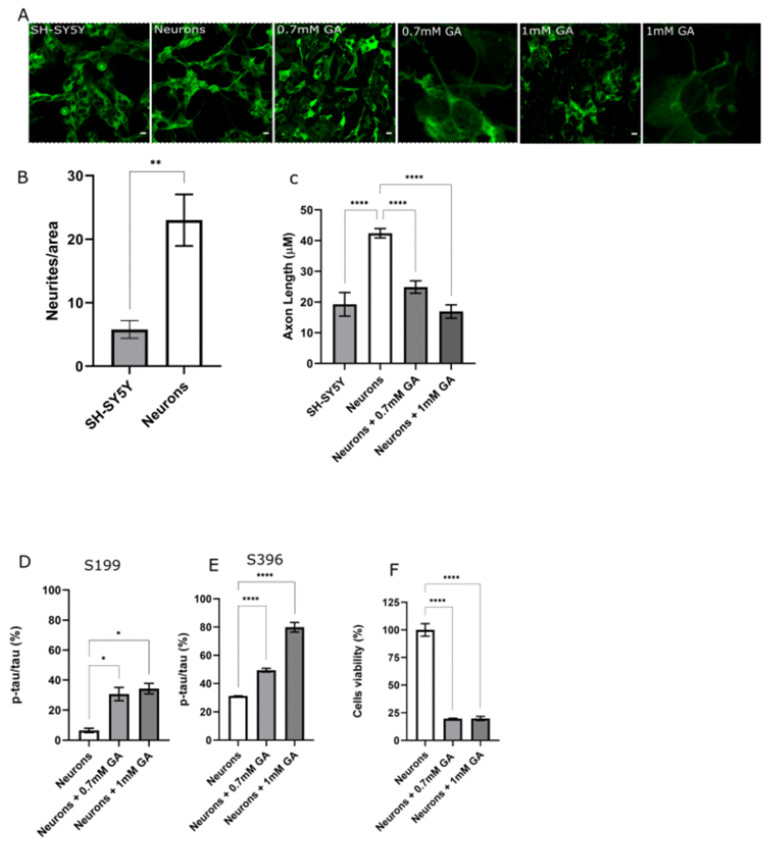
Characterization and validation of novel AD cell model (**A**) Confocal images of SH-SY5Y cells, SH-SY5Y-derived neurons, and SH-SY5Y neurons treated with 0.7 mM GA or 1 mM GA, and zoom-in on degenerating neurites. Scale bar 100 μM. (**B**) Neurite density quantification of SH-SY5Y cells and SH-SY5Y-derived neurons. Mann–Whitney test was used to compare differences between different groups. (**C**) Neurite length quantification of SH-SY5Y cells, SH-SY5-derived neurons, and SH-SY5Y-derived neurons following GA treatment at different concentrations. Ordinary one-way ANOVA followed by Tukey’s post-hoc test was used to compare differences between different groups. (**D**) Quantification of phosphorylation ratio pTau/tTau on S199 of SH-SY5Y-differentiated neurons following 0.7 mM GA or 1 mM treatment. ANOVA followed by Tukey’s post-hoc test was used to compare differences between different groups. (**E**) Quantification of phosphorylation ratio pTau/tTau on S396 of SH-SY5Y-differentiated neurons following 0.7 mM GA or 1 mM GA treatment. ANOVA followed by Tukey’s post-hoc test was used to compare differences between different groups. (**F**) Cell viability of SH-SY5Y-differentiated neurons following GA treatment at different concentrations. Ordinary one-way ANOVA followed by Tukey’s post-hoc test was used to compare differences between different groups. Data are presented as mean ± SEM. *n* = 3. * *p* < 0.05; ** *p* < 0.01; **** *p* < 0.0001.

To develop a neuron-like model of AD bearing Tau hyperphosphorylation, the differentiation of SH-SY5Y to neurons was firstly performed as described by Shipley et al. [26]. SH-SY5Y-derived neurons were subsequently imaged by confocal microscopy (Figure 1A,B) to compare morphological differences. Differentiated neurons exhibited a significant increment in neurite density and neurite length when compared to undifferentiated SH-SY5Y cells (Figure 1A,B).

After validating the neuronal differentiation of SH-SY5Y cells, we investigated whether GA treatment for 24 h would result in any AD defects in differentiated neurons, as described by Koriyama in undifferentiated SH-SY5Y cells [33]. Confocal imaging, and subsequent axon length analysis, showed a significant reduction of neurite length in neurons treated with 0.7 mM GA and 1 mM GA when compared to untreated neurons (Figure 1A,C). In addition to this, an assessment of Tau phosphorylation levels was performed on neurons treated with GA. Tau residue S199 was found to be abnormally phosphorylated following administration of both 0.7 mM GA and 1 mM GA (Figure 1D). The same trend was recorded on S396 residue, recording a significant increment of phosphorylation levels following 0.7 mM GA and 1 mM GA administration (Figure 1E).

Furthermore, we evaluated whether GA treatment, and resulting Tau hyperphosphorylation, would result in any change in neuronal cell viability. To assess neuronal viability, an MTT assay was performed at 24 h after GA administration, with both 0.7 mM GA and 1 mM GA treatment, resulting in a significant decrease in cell viability (Figure 1F).

### 2.2. Novel Compounds Inhibit AChE Enzyme Activity in SH-SY5Y-Differentiated Neurons

Upon validation that GA treatment results in Tau abnormal phosphorylation in SH-SY5Y-derived neurons, we decided to investigate the inhibitory activity of the novel AChE inhibitors that were newly synthesized.

**Figure 2:**
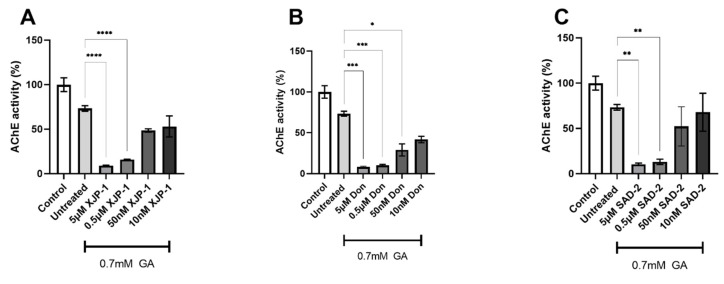

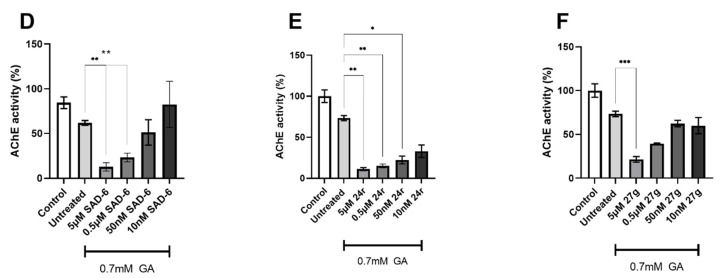
AChE inhibition by novel compounds at different concentrations in 0.7 mM GA-exposed SH-SY5Y-derived neurons. (**A**) Inhibitory activity of compound XJP-1 in 0.7 mM GA-exposed neurons. (**B**) Inhibitory activity of compound Donepezil in 0.7 mM GA-exposed neurons. (**C**) Inhibitory activity of compound SAD-2 in 0.7 mM GA-exposed neurons. (**D**) Inhibitory activity of compound SAD-6 in 0.7 mM GA-exposed neurons. (**E**) Inhibitory activity of compound **24r** in 0.7 mM GA-exposed neurons. (**F**) Inhibitory activity of compound **27g** in 0.7 mM GA-exposed neurons. Ordinary one-way ANOVA followed by Tukey’s post-hoc test was used to compare differences between different groups. Data are presented as mean ± SEM. *n* = 3. * *p* < 0.05; ** *p* < 0.01; *** *p* < 0.001; **** *p* < 0.0001.

In 0.7 mM GA-exposed neurons we recorded a significant inhibition of AChE activity by compound XJP-1 at a concentration up to 0.5 µM, with the same trend being scored by all other AChE inhibitors investigated (Figure 2A–E). However, Donepezil and compound **24r** showed a significant inhibition at 50 nM concentration as well in 0.7 mM GA-treated neurons (Figure 2B,E).

An investigation of dual AChE/GSK-3 inhibitor **27g** showed a significant inhibition at the 5 µM concentration only (Figure 2F).

**Figure 3:**
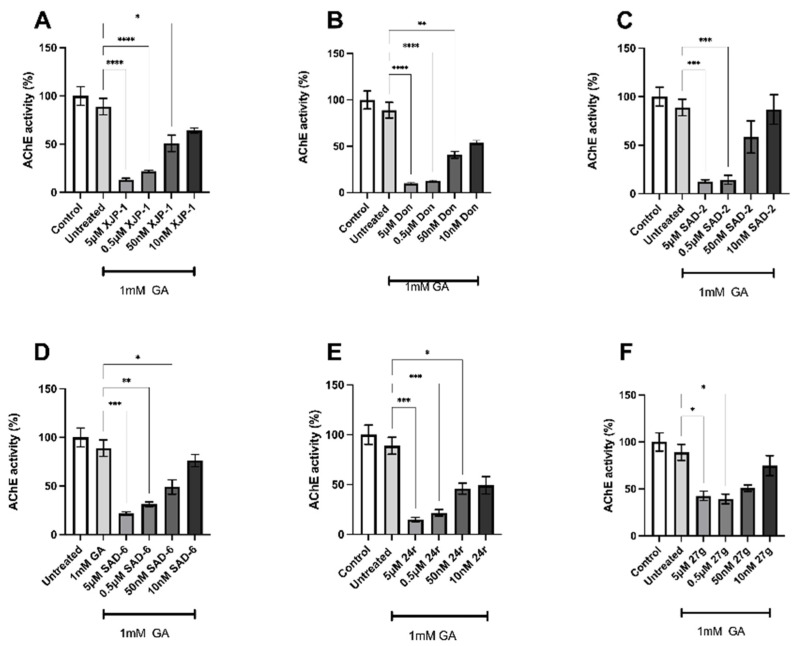
AChE inhibition by novel compounds at different concentrations in 1 mM GA-exposed SH-SY5Y-derived neurons. (**A**) Inhibitory activity of compound XJP-1 in 1 mM GA-exposed neurons. (**B**) Inhibitory activity of compound Donepezil in 1 mM GA-exposed neurons. (**C**) Inhibitory activity of compound SAD-2 in 1 mM GA-exposed neurons. (**D**) Inhibitory activity of compound SAD-6 in 1 mM GA-exposed neurons. (**E**) Inhibitory activity of compound **24r** in 1 mM GA-exposed neurons. (**F**) Inhibitory activity of compound **27g** in 1 mM GA-exposed neurons. Ordinary one-way ANOVA followed by Tukey’s post-hoc test was used to compare differences between different groups. Data are presented as mean ± SEM. *n* = 3. * *p* < 0.05; ** *p* < 0.01; *** *p* < 0.001; **** *p* < 0.0001.

A study on 1 mM GA-exposed neurons showed a decrease in AChE activity following treatment with all inhibitors tested at a concentration up to 50 nM, with the exception of SAD-2 (Figure 3A–F).

### 2.3. AChE Inhibition Prevents GA-Induced Tau Phosphorylation on S396 but Not on S199

**Figure 4:**
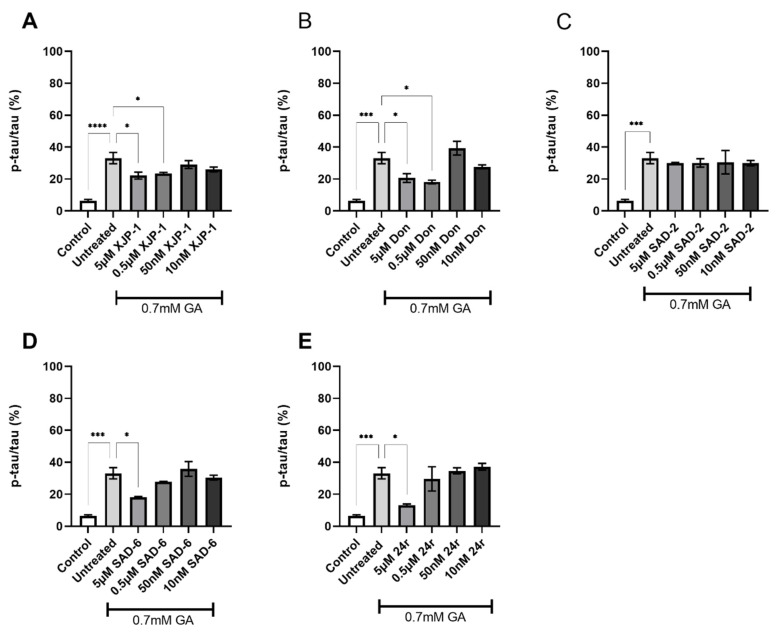
Tau S199 phosphorylation levels in 0.7 mM GA-exposed neurons after treatment with novel AChE inhibitors. (**A**) Quantification of phosphorylation ratio pTau/tTau on S199 of 0.7 mM GA-treated SH-SY5Y-differentiated neurons after XJP-1 treatment. (**B**) Quantification of phosphorylation ratio pTau/tTau on S199 of 0.7 mM GA-treated SH-SY5Y-differentiated neurons after Donepezil treatment. (**C**) Quantification of phosphorylation ratio pTau/tTau on S199 of 0.7 mM GA-treated SH-SY5Y-differentiated neurons after SAD-2 treatment. (**D**) Quantification of phosphorylation ratio pTau/tTau on S199 of 0.7 mM GA-treated SH-SY5Y-differentiated neurons after SAD-6 treatment. (**E**) Quantification of phosphorylation ratio pTau/tTau on S199 of 0.7 mM GA-treated SH-SY5Y-differentiated neurons after **24r** treatment. Kruskal–Wallis test followed by Dunn’s post-hoc was used to compare the differences between different groups. Data are expressed as mean ± SEM, *n* = 3 with at least 5 repetitions per experiment,* *p* < 0.05; *** *p* < 0.001, **** *p* < 0.0001.

After validating the inhibitory activity of the novel compounds on the AChE enzyme (Figure 2 and Figure 3), we investigated whether GA-induced Tau hyperphosphorylation could be prevented by AChE inhibition exerted by novel compounds. The exposure of neuronal cells to 0.7 mM GA dramatically increased Tau phosphorylation levels on S199 (Figure 1D), whilst AChE inhibitor XJP-1, at 5 µM and 0.5 µM concentrations, significantly reduced Tau hyperphosphorylation on S199 in neurons treated with 0.7 mM GA (Figure 4A), with the same trend being followed by the control AChE inhibitor Donepezil (Figure 4B). On the other hand, treatment with novel AChE inhibitors SAD-2 did not reduce S199 phosphorylation in 0.7 mM GA-exposed SH-SY5Y-differentiated neurons (Figure 4C), whilst compounds SAD-6 and **24r** recorded a significant reduction of S199 abnormal phosphorylation when administrated at a 5 µM concentration only (Figure 4D,E). In addition, for SH-SY5Y-differentiated neurons treated with 1 mM GA, none of the AChE inhibitors tested, including Donepezil, showed a significant reduction of Tau phosphorylation on S199 (Figure S1A–E).

**Figure 5:**
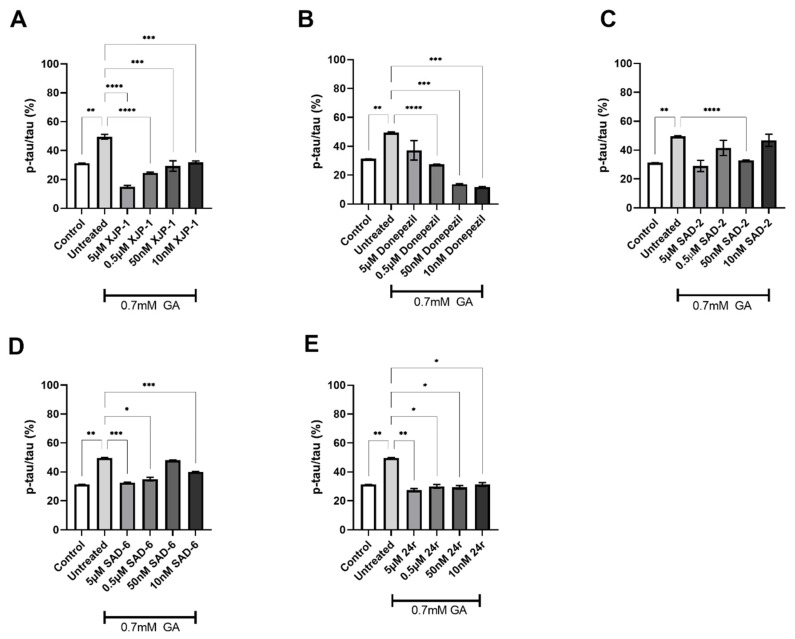
Tau S396 phosphorylation levels in 0.7 mM GA-exposed neurons after treatment with novel AChE inhibitors. (**A**) Quantification of phosphorylation ratio pTau/tTau on S396 of 0.7 mM GA-treated SH-SY5Y-differentiated neurons after XJP-1 treatment. (**B**) Quantification of phosphorylation ratio pTau/tTau on S396 of 0.7 mM GA-treated SH-SY5Y-differentiated neurons after Donepezil treatment. (**C**) Quantification of phosphorylation ratio pTau/tTau on S396 of 0.7 mM GA-treated SH-SY5Y-differentiated neurons after SAD-2 treatment. (**D**) Quantification of phosphorylation ratio pTau/tTau on S396 of 0.7 mM GA-treated SH-SY5Y-differentiated neurons after SAD-6 treatment. (**E**) Quantification of phosphorylation ratio pTau/tTau on S396 of 0.7 mM GA-treated SH-SY5Y-differentiated neurons after **24r** treatment. Kruskal–Wallis test followed by Dunn’s post-hoc was used to compare the differences between different groups. Data are expressed as mean ± SEM, *n* = 3 with at least 5 repetitions per experiment,* *p* < 0.05; ** *p* < 0.01, *** *p* < 0.001, **** *p* < 0.0001.

To further assess the efficacy of the novel compounds in preventing Tau abnormal phosphorylation, another residue, S396, was examined. In neurons treated with 0.7 mM GA, all therapies under study showed a potent effect in reducing S396 phosphorylation, at all concentrations tested (Figure 5A–E).

**Figure 6:**
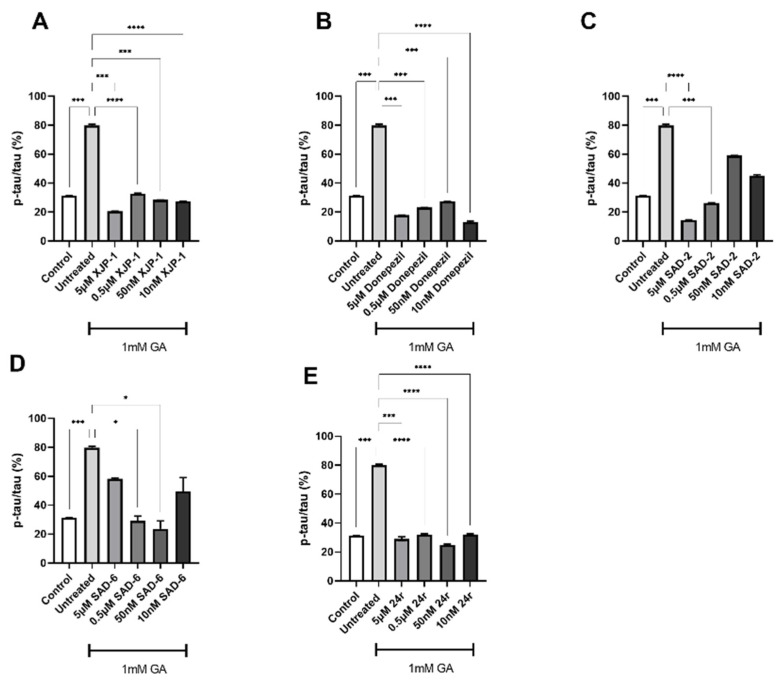
Tau S396 phosphorylation levels in 1 mM GA-exposed neurons after treatment with novel AChE inhibitors. (**A**) Quantification of phosphorylation ratio pTau/tTau on S396 of 1 mM GA-treated SH-SY5Y-differentiated neurons after XJP-1 treatment. (**B**) Quantification of phosphorylation ratio pTau/tTau on S396 of 1 mM GA-treated SH-SY5Y-differentiated neurons after Donepezil treatment. (**C**) Quantification of phosphorylation ratio pTau/tTau on S396 of 1 mM GA-treated SH-SY5Y-differentiated neurons after SAD-2 treatment. (**D**) Quantification of phosphorylation ratio pTau/tTau on S396 of 1 mM GA-treated SH-SY5Y-differentiated neurons after SAD-6 treatment. (**E**) Quantification of phosphorylation ratio pTau/tTau on S396 of 1 mM GA-treated SH-SY5Y-differentiated neurons after **24r** treatment. Kruskal–Wallis test followed by Dunn’s post-hoc was used to compare the differences between different groups. Data are expressed as mean ± SEM, *n* = 3 with at least 5 repetitions per experiment, * *p* < 0.05; *** *p* < 0.001, **** *p* < 0.0001.

An overlapping trend was found in neurons treated with 1 mM GA, with all the novel compounds inducing a significant reduction of phosphorylation levels on Tau S396, at a concentration as low as 10 nM, with the exception of SAD-6 (Figure 6A–E).

### 2.4. Dual AChE/GSK-3 Inhibitor **27g** Modulates Tau Phosphorylation on Both S199 and S396

**Figure 7:**
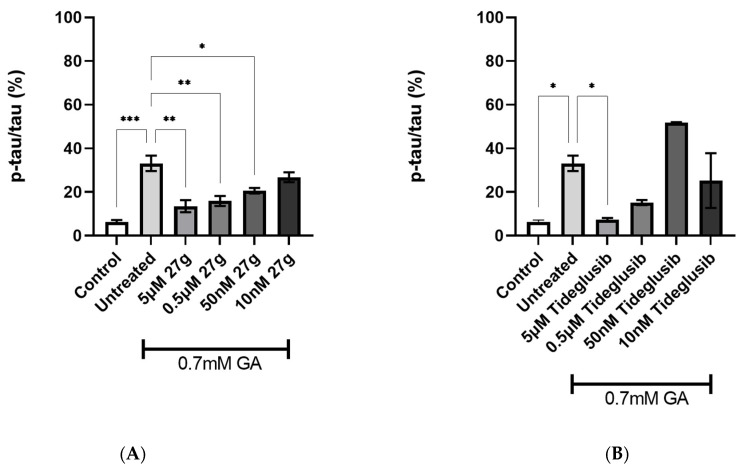
Reduction of Tau S199 phosphorylation after **27g** treatment in 0.7 mM GA-exposed neurons. (**A**) Quantification of phosphorylation ratio pTau/tTau on S199 of 0.7 mM GA-treated SH-SY5Y-differentiated neurons after **27g** treatment. (**B**) Quantification of phosphorylation ratio pTau/tTau on S199 of 0.7 mM GA-treated SH-SY5Y-differentiated neurons after Tideglusib treatment. Kruskal–Wallis test followed by Dunn’s post-hoc was used to compare the differences between different groups. Data are expressed as mean ± SEM, *n* = 3 with at least 5 repetitions per experiment, * *p* < 0.05; ** *p* < 0.01, *** *p* < 0.001.

To study the effects of treatment with dual inhibitor AChE/GSK-3 **27g**, Tau phosphorylation on S199, in 0.7 mM GA-treated neurons, was assessed. The novel compound showed a significant reduction of S199 phosphorylation levels when compared to the untreated 0.7 mM GA neurons, in a dose-dependent manner up to 50 nM concentration (Figure 7A), whilst commercial GSK-3 inhibitor Tideglusib reduced S199 phosphorylation at the 5 µM concentration only (Figure 7B). However, the novel dual inhibitor was not able to significantly reduce S199 phosphorylation in neurons exposed to 1 mM GA (Figure S2A), despite the GSK-3 inhibitor Tideglusib successfully reducing S199 phosphorylation at 5 µM and 0.5 µM concentrations (Figure S2B).

**Figure 8:**
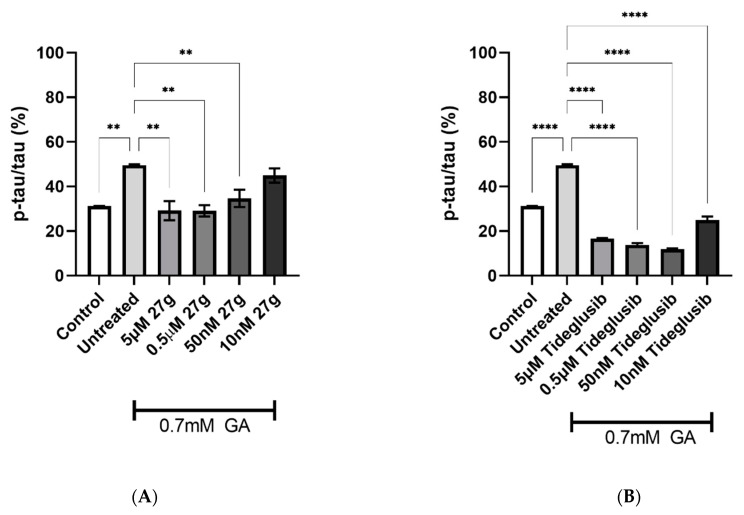
Reduction of Tau S396 phosphorylation after **27g** treatment in 0.7 mM GA-exposed neurons. (**A**) Quantification of phosphorylation ratio pTau/tTau on S396 of 0.7 mM GA-treated SH-SY5Y-differentiated neurons after **27g** treatment. (**B**) Quantification of phosphorylation ratio pTau/tTau on S396 of 0.7 mM GA-treated SH-SY5Y-differentiated neurons after Tideglusib treatment. Kruskal–Wallis test followed by Dunn’s post-hoc was used to compare the differences between different groups. Data are expressed as mean ± SEM, *n* = 3 with at least 5 repetitions per experiment; ** *p* < 0.01, **** *p* < 0.0001.

**Figure 9:**
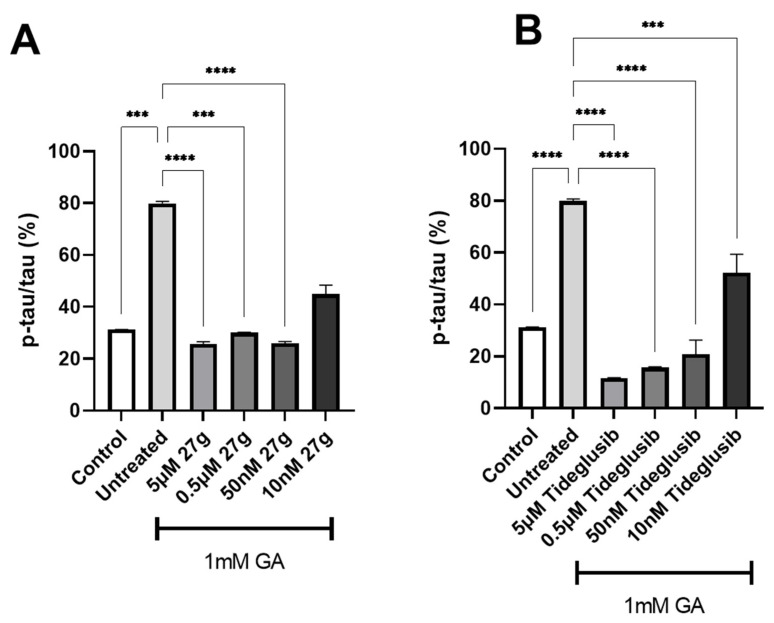
Reduction of Tau S396 phosphorylation after **27g** treatment in 1 mM GA-exposed neurons. (**A**) Quantification of phosphorylation ratio pTau/tTau on S396 of 1 mM GA-treated SH-SY5Y-differentiated neurons after **27g** treatment. (**B**) Quantification of phosphorylation ratio pTau/tTau on S396 of 1 mM GA-treated SH-SY5Y-differentiated neurons after Tideglusib treatment. Kruskal–Wallis test followed by Dunn’s post-hoc was used to compare the differences between different groups. Data are expressed as mean ± SEM, *n* = 3 with at least 5 repetitions per experiment, *** *p* < 0.001, **** *p* < 0.0001.

To further characterize the efficacy of compound **27g** in reducing Tau hyperphosphorylation, residue S396 was examined. A significant decrease in S396 phosphorylation levels was recorded, up to a 50 nM concentration, in 0.7 mM GA-exposed neurons treated with compound **27g**, with Tideglusib scoring similar results (Figure 8A,B). Moreover, inhibition of both AChE and GSK-3 enzymes resulted in a significant reduction for all concentrations tested of compound **27g** in 1 mM GA-exposed neurons, with the same results being scored by the control drug Tideglusib (Figure 9A,B).

### 2.5. Dual AChE/GSK-3 Inhibitor **27g** Prevents Tau-Induced Neurodegeneration and Protects from Neuronal Cell Death

**Figure 10:**
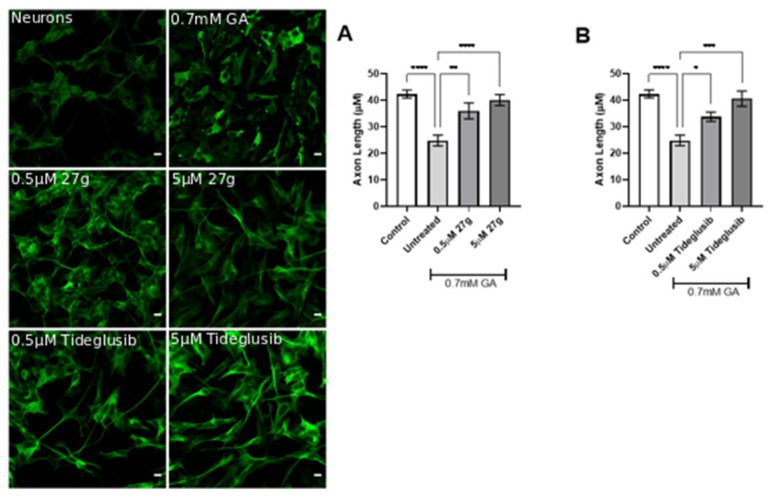
Dose-dependent amelioration of neurite morphology by compound **27g** in 0.7 mM GA-exposed neurons. (**A**) Neurite length quantification of 0.7 mM GA-treated SH-SY5Y-differentiated neurons after **27g** treatment. Scale bar 100 μM. (**B**) Neurite length quantification of 0.7 mM GA-treated SH-SY5Y-differentiated neurons after Tideglusib treatment. Ordinary one-way ANOVA followed by Tukey’s post-hoc test was used to compare differences between different groups. Data are presented as mean ± SEM, *n* = 3 with at least 5 repetitions per experiment, * *p* < 0.05; ** *p* < 0.01; *** *p* < 0.001; **** *p* < 0.0001.

During AD progression, the formation of aggregated forms of hyperphosphorylated Tau leads to the disruption of neuronal morphology, with jeopardized neural architecture resulting in a loss of brain circuits and functions. Thus, the prevention of neurite degeneration is a fundamental test for a potential AD therapy. We have exploited confocal microscopy to investigate the neuronal morphology in the presence of any ameliorations at 24 h after incubation with 0.7 mM GA and compound **27g** at various concentrations. We found that compound **27g** significantly prevented neurite shortening in a dose-dependent manner and exhibited more potent effects than Tideglusib at the same concentrations (Figure 10A,B).

**Figure 11:**
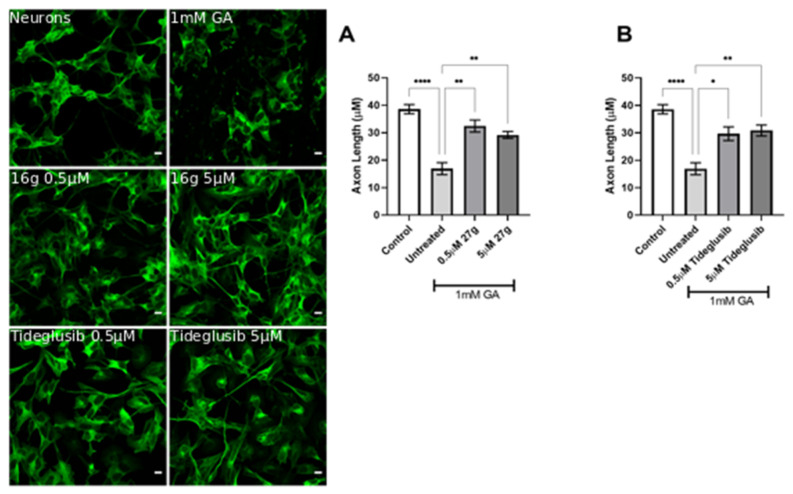
Amelioration of neurite morphology by compound **27g** in 1 mM GA-exposed neurons. (**A**) Neurite length quantification of 1 mM GA-treated SH-SY5Y-differentiated neurons after **27g** treatment. Scale bar 100 μM. (**B**) Neurite length quantification of 1 mM GA-treated SH-SY5Y-differentiated neurons after Tideglusib treatment. Ordinary one-way ANOVA followed by Tukey’s post-hoc test was used to compare differences between different groups. Data are presented as mean ± SEM, *n* = 3 with at least 5 repetitions per experiment,* *p* < 0.05; ** *p* < 0.01; **** *p* < 0.0001.

The inhibition of both AChE and GSK-3 by compound **27g** confirmed the positive trend by preventing neurite shortening in 1 mM GA-treated neurons as well, overlapping Tideglusib results (Figure 11A,B). In addition to this, we observed an improvement of cell viability at both concentrations of GA following administration of **27g** or Tideglusib (Figure S3A–D). Conversely, treatment with AChE inhibitors resulted in variable results in preventing neurodegeneration, both in terms of morphology protection (Figures S4 and S5) and cell viability (Figures S6 and S7).

## 3. Discussion

In AD, the regulation of multiple pathways involved in the pathogenesis and progression of the disease is the main target for a potential disease-modifying therapy. The novel compound **27g** aims to do so by inhibiting both enzymes of AChE and GSK-3 simultaneously, with its structure designed using Tacrine and Pyrimidone as moieties [37]. The exploitation of a cell model that well recapitulates AD neuronal features is an important approach to better characterize newly synthesized compounds for AD treatment. We have successfully combined two different protocols, developed and validated a neuronal cell model of AD bearing Tau hyperphosphorylation (Shipley 2016; Koriyama et al., 2015). In particular, using GA to inhibit glycolysis, and increase AGEs level, well recapitulates the AD human features, in which the accumulation of AGEs and subsequent activation of the RAGE receptor is often described, leading to the activation of the amyloidogenic pathway and Tau hyperphosphorylation [1,38,39,40,41]. The accumulation of AGEs as a consequence of GA treatment results in Tau disease, as seen in diabetes, of which the pathology is often linked to the development of AD. The model developed recapitulates the neuronal features of AD well, with GA treatment leading to abnormal phosphorylation of Tau protein on different residues and neurite degeneration, along with reduced cell viability. This model is of peculiar relevance as it offers a novel platform to further study RAGE signalling, which has often been linked to AD development [8]. RAGE is an immunoglobulin superfamily receptor capable of binding AGEs, Amyloid beta, and other ligands. In a study involving both SH-SY5Y and hippocampal primary neurons, the application of AGEs induced a dose-dependent increase on Tau hyperphosphorylation on multiple residues, including S199, S396, S404 [42]. These results overlap our findings obtained after GA treatment, showing an increase in aberrant phosphorylation in Tau S199 and S396. The AChE inhibitory compounds (XJP-1, SAD-2, and SAD-6) with a 1-benzyl-pyridin-1-ium bromide backbone structure and with the pharmacophore skeleton 1-benzyl-pyridin-1-ium (Table 1) were evaluated with this cell model. Among these compounds, the in silico predicted potency order is SAD-2 > XJP-1 > SAD-6, suggesting the influence of these substituents is Se > O > S, which did not reflect a similar result upon completion of biological assays. In addition, another sulfone analogue of Donepezil, compound **24r**, was screened by this cell model, and the results showed that the **24r** structure backbone is best compared to XJP or SAD, but again failed to produce any further beneficial effects when compared to the other AChE inhibitors. The dual AChE/GSK-3 inhibitor, compound **27g**, with a Tacrine–Pyrimidone skeleton, was active in the cell model, and also displayed potent in vivo anti-AD efficacy. The assessment of dual AChE/GSK-3 inhibitor **27g** showed promising results, with overlapping results obtained by the commercially available GSK-3 inhibitor Tideglusib. Therefore, the simultaneous inhibition of AChE and GSK-3 improves the efficacy in vivo [37]. On the other hand, assessment of different single-targeting AChE inhibitors did not result in a homogenous comparable outcome, despite significant inhibition of the enzyme recorded in vitro [43], suggesting that inhibition of the AChE enzyme may modulate different AD pathways involved in the Tau hyperphosphorylation triggered by GA, but in a non-specific manner derived by the distribution and concentration of different acetylcholine receptors. Finally, for several compounds, a modest inhibition of AChE enzyme still resulted in a significant amelioration of the parameters analyzed, further suggesting that inhibition of the AChE enzyme results in a response controlled by the pathways activated by specific acetylcholine receptors.

## 4. Materials and Methods

### 4.1. Reagents 

AChE inhibitor Donepezil and GSK-3β inhibitor tideglusib were purchased from Sigma-Aldrich Ltd (St. Louis, MO, USA). Drug candidates of novel AChE inhibitors XJP-1, SAD-2, SAD-6, and **24r** and **27g** were gifted by Prof. Jinyi Xu (China Pharmaceutical University, Nanjing, China). All drug compounds were dissolved in DMSO to make a stock solution with a final concentration of 1 mM and stored at −20 °C. The compound structure is outlined in Table 1.

### 4.2. SH-SY5Y Culture and Neuronal Differentiation

SH-SY5Y cells were cultured on different types of media, described in Table 2, and differentiated as described by Shipley with some modifications [26]. Briefly, SH-SY5Y cells were plated with Basic Growth media and allowed to reach 70% confluency. Subsequently, the medium was changed to Differentiation Media-1 and replaced every 48 h for the following 7 days. Cells were then split 1:1 and moved into fresh flasks/dishes and Differentiation Media-2 was added. Differentiation Media-2 was then replaced every 48 h for the following 4 days. Subsequently, the medium was changed for Differentiation Media-3 and replaced every 48 h for the following 7 days. After this period, SH-SY5Y-derived neurons were used for the subsequent assays and analysis.

### 4.3. Glyceraldehyde Induced Tau Hyperphosphorylation

In order to induce Tau hyperphosphorylation, differentiated SH-SY5Y were treated with either 0.7 mM or 1 mM GA for 24 h as previously described [33]. After treatment with GA for 24 h, cells were used for downstream analysis.

### 4.4. Immunostaining Cell Culture

SH-SY5Y cells and differentiated SH-SY5Y cells were fixed in 4% PFA for 10 min at room temperature. Each sample was washed three times with 0.1% PBS-T for 2 min. Following fixation, cells were incubated at room temperature for 2 h in 5% NGS-T blocking solution and incubated overnight at +4 °C with the mouse monoclonal anti-tubulin III primary antibody (#ab179513, Abcam plc., Camabridge, UK) at a final concentration of 1:1000. The primary antibody was then removed, and each sample was washed three times with 0.1% PBS-T. Rabbit anti-mouse secondary antibody Alexa Fluor 488 (#ab150113, Abcam plc., Cambridge, UK) was added at a final 1:2000 concentration and left to incubate at RT for 1 h. The secondary antibody was removed, and each sample was washed three times with 0.1% PBS-T. Samples were then mounted on to glass slides, using FluoroGel Mounting media (Genetex, Irvine, CA, USA). Samples were finally imaged by confocal microscopy within 24 h.

### 4.5. Confocal Microscopy

Cell images were acquired using Zeiss880 Confocal Microscope (Zeiss, Jena, Germany). Laser power was set at 4%, gain 650, digital offset at 350. Acquisition speed was set at 1.3 m, averaging two times per image. Z-stack images at distance of 5 µm were acquired. Analysis of confocal images was performed using ImageJ v2 plug-in NeuronJ v1. Three more random areas were analyzed. A threshold mask was applied to visualize the axon only and not the cell body. Subsequently, the measurement of the axon length was taken, and the distance between coordinates was measured with NeuronJ.

### 4.6. pTAU Quantification

Phosphorylation levels of Tau protein on Serine 199 (S199) and Serine 396 (S396) were quantified using Enzyme Linked Immunosorbent Assay (ELISA) methodology. Following the manufacturer recommended protocol, ELISA kits KHB7041, KHB7031, and KHB0041 (ThermoFisher Scientific, Waltham, MA, USA) were used to quantify phosphorylated Tau S199, phosphorylated S396, and total Tau, respectively. Phosphorylation percentage was obtained for the analyzed residues by normalization against total Tau.

### 4.7. AChE Activity Assay (Ellman’s Method)

AChE enzymatic activity was assessed using AChE assay kit (#ab138871, Abcam plc.) following the manufacturer’s instructions. A total of 10^5^ per well were seeded into a clear 96-well plate. After appropriate differentiation and treatment, cell culture medium was removed, and 100 µL of lysis buffer was added into each well and left to incubate for 15 min at room temperature (RT). Subsequently, 50 µL of acetylthiocholine reaction mixture (1× assay buffer, 1× DTNB stock solution, 1× acetylthiocholine stock solution) was added to each well and samples were left to incubate for 30 min at RT. Samples were then analyzed using a 96-well microplate reader at OD= 410 ± 5 nm. In order to avoid false positives given by butyrylcholinesterase activity, the specific AChE inhibitor Donepezil hydrochloride was used as control.

### 4.8. Cell Viability Assay

A total of 10^5^ per well were seeded into a 96-well plate. After SH-SY5Y differentiation, cells were treated with either 0.7 mM or 1 mM GA, respectively. Along with GA treatment, an appropriate concentration of the compound to be tested was added. After 24 h, 25 μL of 5 mg/mL Methylthiazolyldiphenyl-tetrazolium bromide (MTT) (#M2128-100MG, Sigma-Aldrich, St. Louis, MO, USA) was added into each well without removing cell culture media and incubated at 37 °C with 5% CO_2_ for 2 h. Subsequently, 100 μL of lysing buffer (50% SDS solution, 25% DMF, 25% demineralized water) was added. After an overnight incubation (20 h) at 37 °C, the OD at 490 nm was measured using 96-well plate reader. The medium/MTT/lysing buffer incubated under the same conditions was used as the control.

### 4.9. Data Analysis

Statistical analysis was performed using GraphPad Prism 9 software. Data obtained were firstly tested for normality using the Shapiro–Wilk test. The Kruskal–Wallis test followed by Dunn’s post-hoc was used to compare the differences between three or more groups in non-normally distributed data. The ANOVA test was used to compare the differences between three or more groups of normally distributed samples. Each experiment was performed in triplicate and all results are presented as mean ± standard error of the mean or mean ± standard deviation. Results with a *p*-value < 0.05 were considered as statistically significant.

## 5. Conclusions

In the present work, we provided an upgraded version of the Tau hyperphosphorylation model initially designed by Koryiama [33] on undifferentiated SH-SY5Y. We screened different AChE inhibitors along with the already reported **24r** and **27g** compounds [37,43], and for both we expanded the investigation on 1 mM GA-treated cells. The screening has confirmed the variability of AChE inhibitors on modulating AD features, whilst suggesting that a dual GSK-3/AChE inhibition strategy could be a potential route for the development of an AD therapy with potential disease-modifying properties. Our finding provides the basis for further studies in more complex AD models, i.e., AD risk gene-edited neurons or astrocytes, to investigate different pathological features such as amyloid pathology and synapsis activation.

## Figures and Tables

**Table 1:** Structures of novel compounds. Single AChE inhibitors and dual AChE/GSK-3 inhibitor and IUPAC names. (A) XJP-1. (B) SAD-2. (C) SAD-6. (D) **24r**. (E) **27g**.

No.	Compd.	Structures
**A**	**XJP-1**	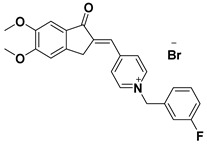
Name: (*E*)-4-((5,6-dimethoxy-1-oxo-1,3-dihydro-2H-inden-2-ylidene)methyl)-1-(3-fluorobenzyl)pyridin-1-ium bromide		
**B**	**SAD-2**	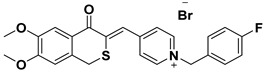
Name: (*Z*)-4-((6,7-dimethoxy-4-oxoisothiochroman-3-ylidene)methyl)-1-(4-fluorobenzyl)pyridin-1-ium bromide		
**C**	**SAD-6**	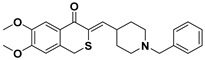
Name: (*Z*)-3-((1-benzylpiperidin-4-yl)methylene)-6,7-dimethoxyisothiochroman-4-one		
**D**	**24r**	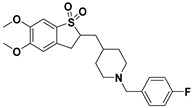
Name: 2-((1-(4-fluorobenzyl)piperidin-4-yl)methyl)-5,6-dimethoxy-2,3-dihydrobenzo[b]thiophene 1,1-dioxide		
**E**	**27g**	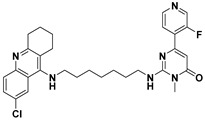
Name: 2-((7-((6-chloro-1,2,3,4-tetrahydroacridin-9-yl)amino)heptyl)amino)-6-(3-fluoropyridin-4-yl)-3-methylpyrimidin-4(3H)-one		

**Table 2:** Cell culture media for SH-SY5Y neuronal differentiation. Breakdown of different cell culture media used for SH-SY5Y differentiation protocol.

Basic Growth Media	Differentiation Media #1	Differentiation Media #2	Differentiation Media #3
EMEM	EMEM	EMEM	Neurobasal
15% hiFBS	2.5% hiFBS	1% hiFBS	20 mM KCl
1x Pen/Strep	1x Pen/Strep	1x Pen/Strep	1x Pen/Strep
2 mM Glutamine	2 mM Glutamine	2 mM Glutamine	2 mM Glutamax
	10 μM RA	10 μM RA	10 μM RA
			50 ng/mL BDNF

## Data Availability

Not applicable.

## Supplementary Materials

The supporting information can be downloaded at: https://www.mdpi.com/article/10.3390/ijms232314794/s1.
