# Supplementary figures and images for: Development of p-Tau Differentiated Cell Model of Alzheimer’s Disease to Screen Novel Acetylcholinesterase Inhibitors
Source: Int J Mol Sci. 2022 Nov 26;23(23):14794. doi: 10.3390/ijms232314794 (PMC9741399; doi:10.3390/ijms232314794)

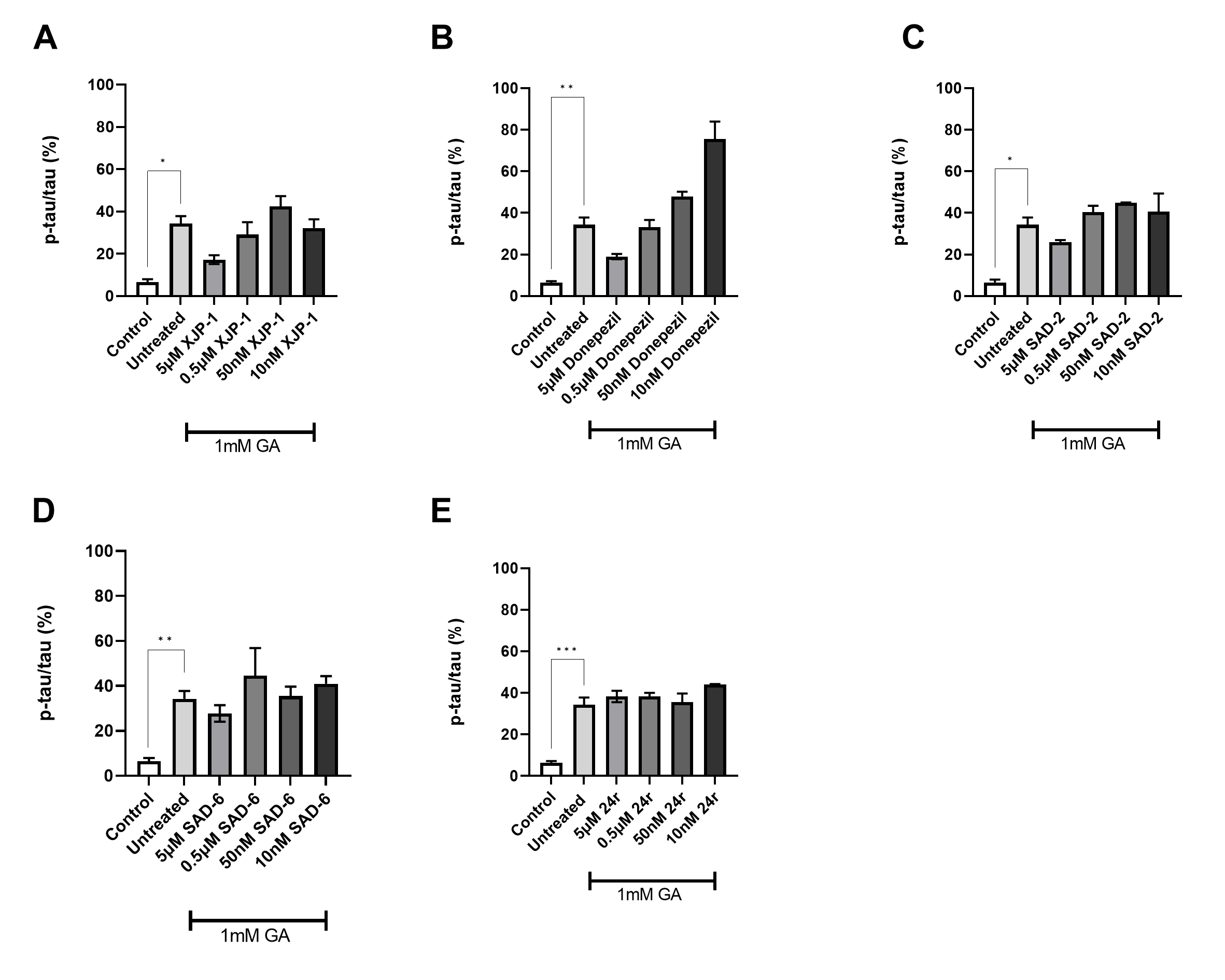

Supplement: Supplementary file 1 [file ijms-23-14794-s001.zip › S1.tif]

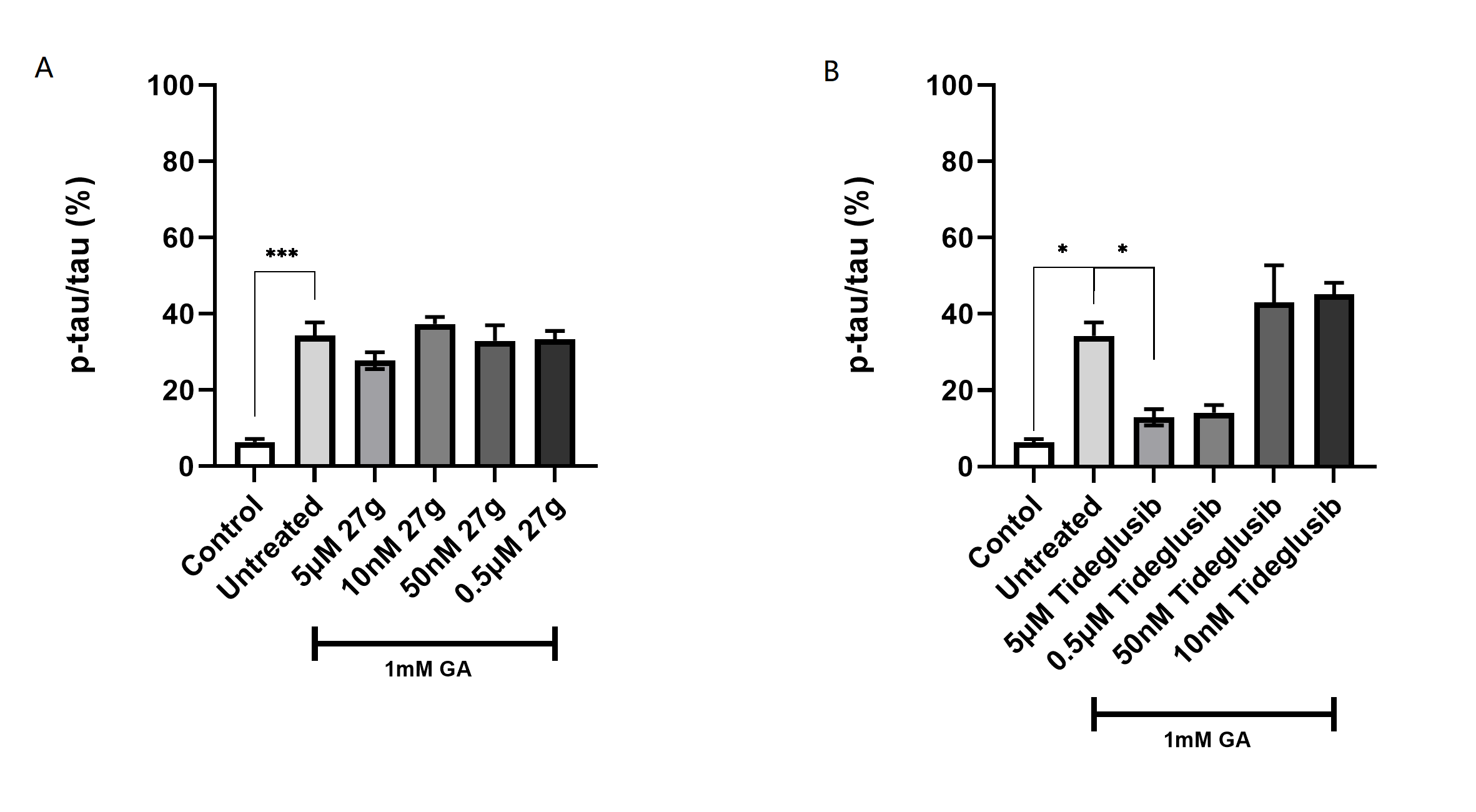

Supplement: Supplementary file 1 [file ijms-23-14794-s001.zip › S2.tif]

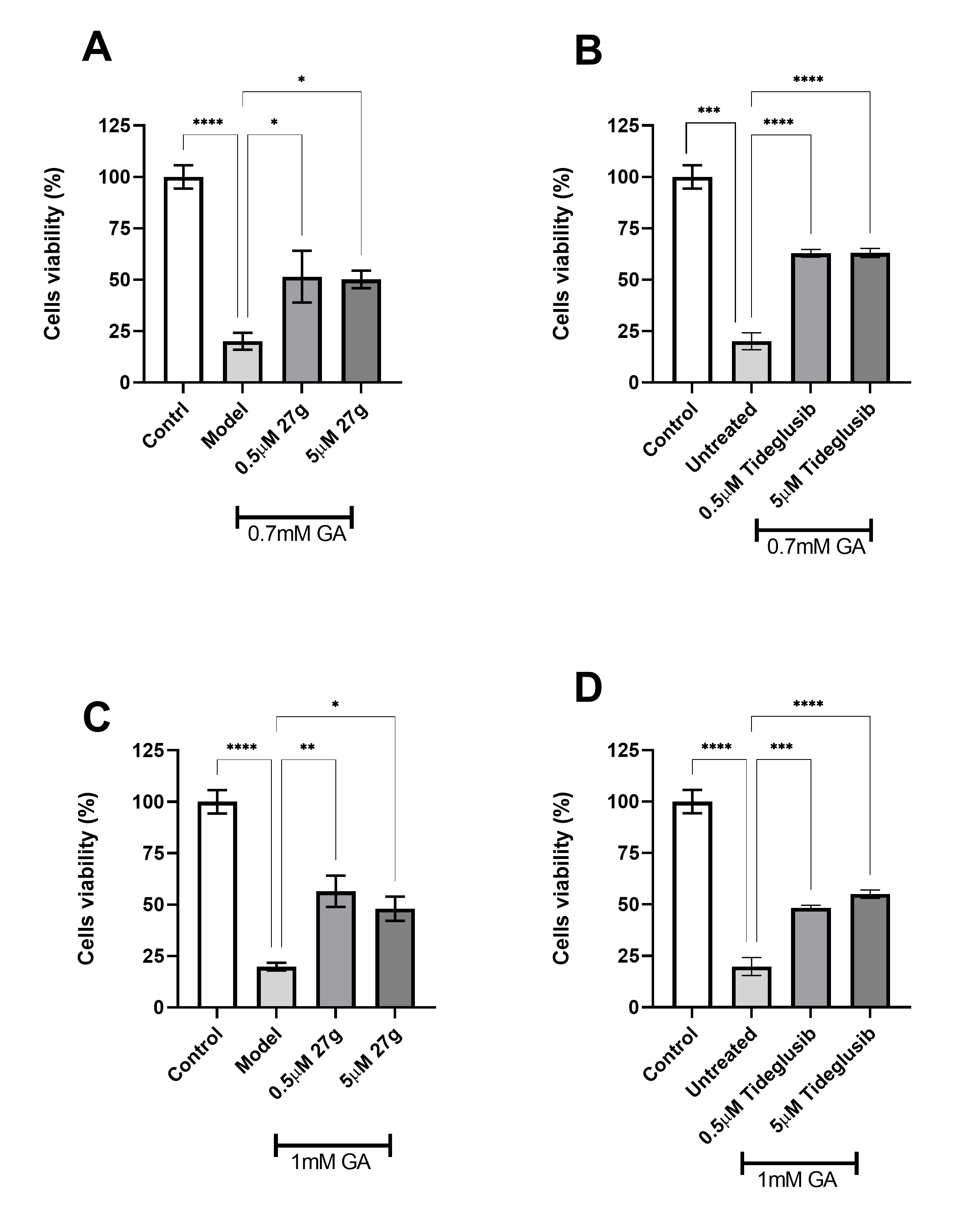

Supplement: Supplementary file 1 [file ijms-23-14794-s001.zip › S3.tif]

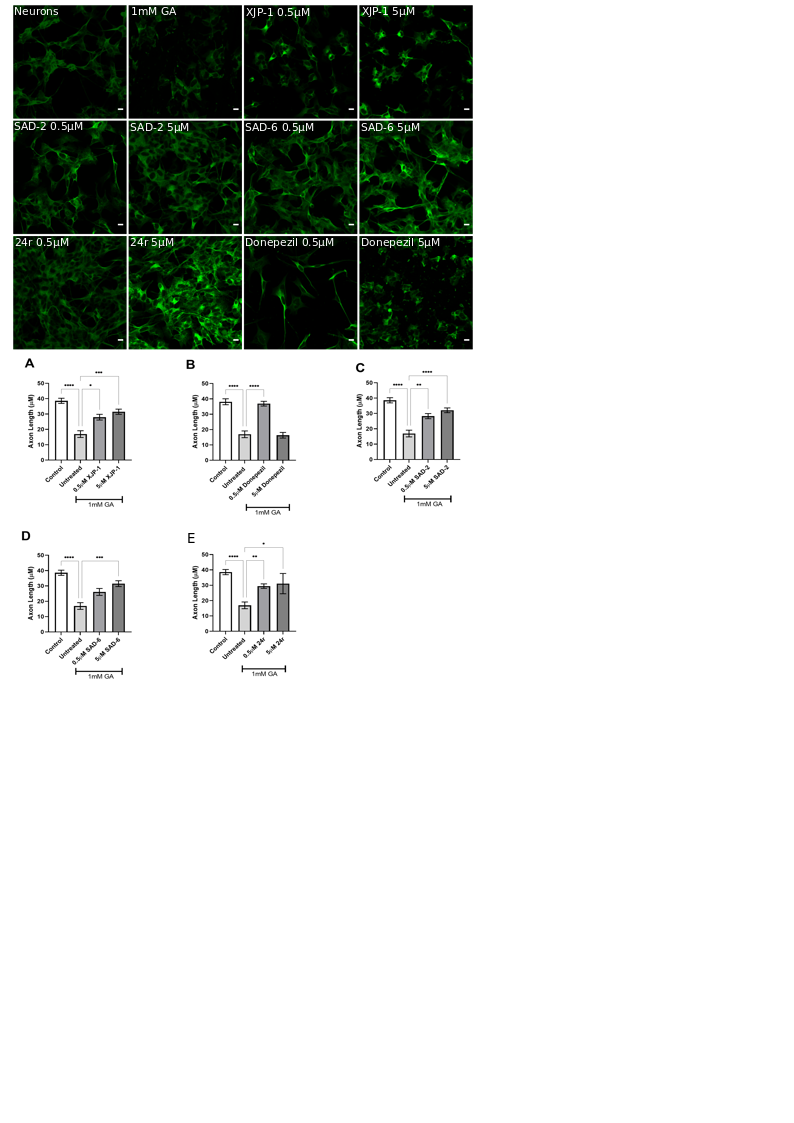

Supplement: Supplementary file 1 [file ijms-23-14794-s001.zip › S4.png]

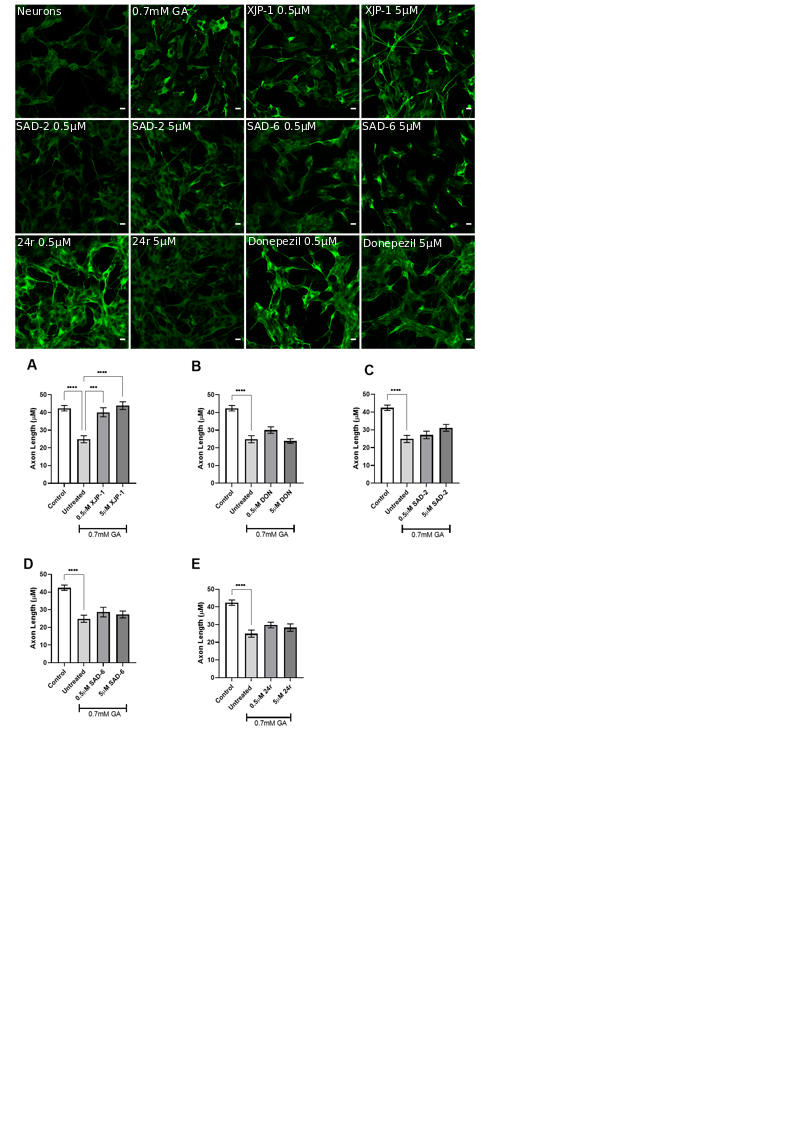

Supplement: Supplementary file 1 [file ijms-23-14794-s001.zip › S5.png]

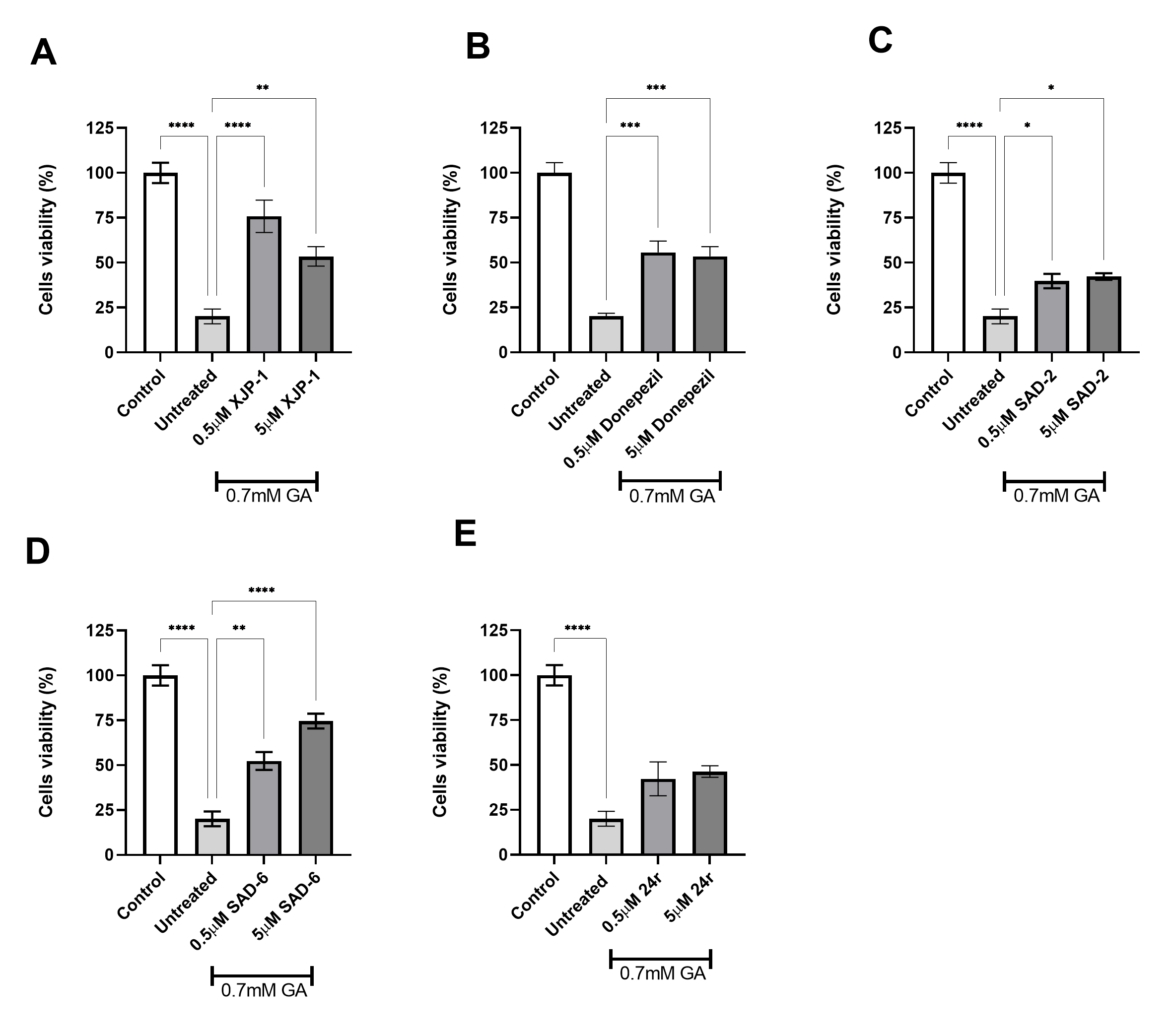

Supplement: Supplementary file 1 [file ijms-23-14794-s001.zip › S6.tif]

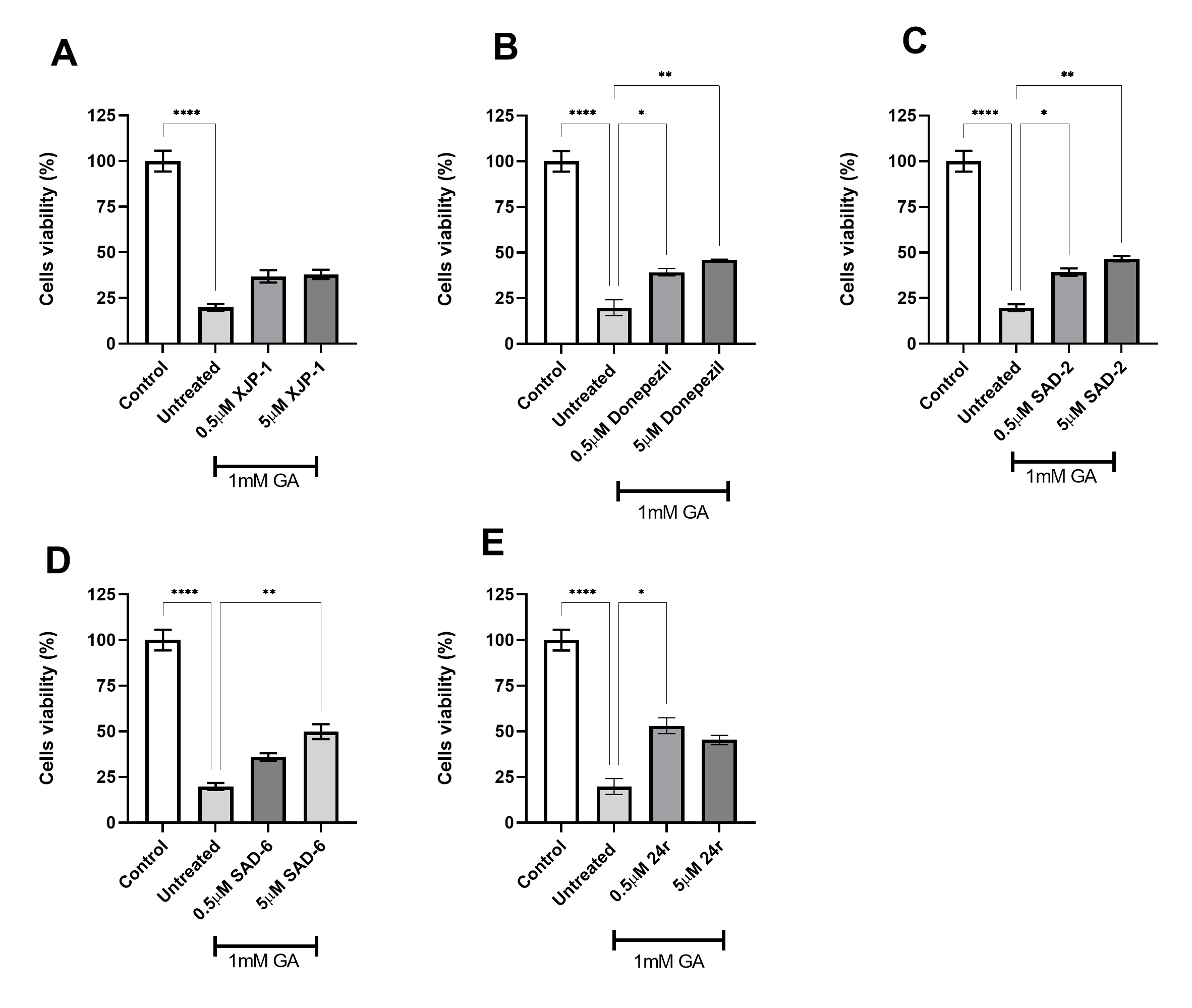

Supplement: Supplementary file 1 [file ijms-23-14794-s001.zip › S7.tif]
